# Cannabidiol, a Non-Psychoactive Cannabinoid Compound, Inhibits Proliferation and Invasion in U87-MG and T98G Glioma Cells through a Multitarget Effect

**DOI:** 10.1371/journal.pone.0076918

**Published:** 2013-10-21

**Authors:** Marta Solinas, Paola Massi, Valentina Cinquina, Marta Valenti, Daniele Bolognini, Marzia Gariboldi, Elena Monti, Tiziana Rubino, Daniela Parolaro

**Affiliations:** 1 Department of Theoretical and Applied Sciences, Biomedical Research Division, Centre of Neuroscience, University of Insubria, Busto Arsizio, Varese, Italy; 2 Department of Pharmacology, Chemotherapy and Toxicology, University of Milan, Milan, Italy; 3 Department of Pharmacology and Toxicology, University of Toronto, Toronto, Canada; Complutense University, Spain

## Abstract

In the present study, we found that CBD inhibited U87-MG and T98G cell proliferation and invasiveness *in vitro* and caused a decrease in the expression of a set of proteins specifically involved in growth, invasion and angiogenesis. In addition, CBD treatment caused a dose-related down-regulation of ERK and Akt prosurvival signaling pathways in U87-MG and T98G cells and decreased hypoxia inducible factor HIF-1α expression in U87-MG cells. Taken together, these results provide new insights into the antitumor action of CBD, showing that this cannabinoid affects multiple tumoral features and molecular pathways. As CBD is a non-psychoactive phytocannabinoid that appears to be devoid of side effects, our results support its exploitation as an effective anti-cancer drug in the management of gliomas.

## Introduction

Malignant gliomas are among the most rapidly growing and devastating neoplasms with poor prognosis. Despite years of research in anti-tumoral therapeutic strategies and aggressive treatments including surgery, radiotherapy, and chemotherapy, these tumors invariably recur and generally lead to death within less than one year from diagnosis. Gliomas rarely metastasize out of the central nervous system, but their aggressive invasion of normal peritumoral tissue makes surgical removal virtually impossible [1]. Among the cellular signals that prompt tumor cells to egress from the tumor mass and are associated with enhanced glioma invasiveness, an important role is played by the high expression levels of matrix metalloproteinases (MMPs), a family of enzymes promoting tissue breakdown and remodelling through the degradation of extracellular matrix (ECM) [2], as well as the expression of several other proteases and factors [3]. Moreover, the up-regulation of many important signaling molecules downstream of the mitogen activated protein kinases MAPK/ERK and the PI3K/Akt pathways appears to be involved in glioma tumorigenesis and invasion associated with the aberrant tumor growth. Therefore, it can be suggested that controlling the expression of all of these factors may represent a promising therapeutic strategy for treating gliomas.

In addition, gliomas invariably develop hypoxic areas within the tumor mass as distinctive feature. Tumor hypoxia is a powerful driving force for malignant progression by activating adaptive transcriptional programs that promote cell survival, invasiveness and tumor angiogenesis [4], thus selecting a subpopulation of tumor cells fit to survive other injuries (including radio- and chemotherapy). Hypoxia-inducible factor-1 (HIF-1), a heterodimeric transcription factor consisting of a hypoxia-inducible α subunit and a constitutively expressed β subunit, has been identified as a master regulator of the hypoxic response [5], [6] with pleiotropic effects: promotion of invasion, angiogenesis, switch to glycolytic metabolism, and up-regulation of cell survival-related molecules. Hence, HIF-1 has emerged as an attractive target for the development of novel antiglioma agents.

Cannabinoids derived from the plant *Cannabis sativa*, as well as their endogenous and synthetic counterparts, are currently receiving attention as potential chemotherapeutic agents [7], [8]. In particular, we previously demonstrated that the non-psychoactive cannabinoid compound cannabidiol (CBD) effectively limits human glioma cell growth, both *in vitro* and *in vivo*, by triggering apoptosis, oxidative stress, inhibition of the lipoxygenase (LOX) pathway and by modulating the endocannabinoid system [9]–[12]. In addition, CBD interferes with angiogenesis associated to tumor growth [13].

Nevertheless, studies exploring the putative anti-invasive properties of CBD in glioma cells are still limited and the molecular mechanisms underlying its effect are poorly understood. Thus, in the present study we aimed at characterizing the antiproliferative/antiinvasive properties of CBD in two different glioma cell lines (U87-MG and T98G cell) and we investigated its ability to interfere with the expression of several proteins specifically involved in tumor growth and spreading. Moreover, we evaluated CBD ability to affect the most relevant pro-tumoral ERK and PI3K/Akt signaling pathways, as well as the expression of the fundamental transcription factor HIF-1α.

## Materials and Methods

### Reagents

Standard chemicals and cell culture reagents were purchased from Sigma Aldrich. CBD was a generous gift from GW Pharma. It was initially dissolved in ethanol to a concentration of 50 mM and stored at −20°C and further diluted in complete tissue culture medium; final ethanol concentration never exceeded 0.05%.

### Cell Culture

The human glioma cell lines U87-MG and T98G were obtained from the American Type Culture Collection. Cells were maintained in DMEM supplemented with 10% heat-inactivated fetal bovine serum (Euroclone), 2% L-glutamine, 1% antibiotic mixture, 1% sodium pyruvate, 1% non-essential aminoacids (Sigma Aldrich), at 37°C in a humidified 5% CO_2_ atmosphere. Cells were seeded in complete medium. After a 24 h incubation, the medium was replaced by serum-free medium (ITSS medium), consisting of DMEM supplemented with 5 µg/ml insulin, 5 µg/ml transferrin, and 5 µg/ml sodium selenite.

### MTT Test

To determine the effects of CBD on cell viability, the MTT colorimetric assay was carried out as previously reported [9]. Briefly, U87-MG glioma cells were seeded in a 96-well flat bottom multiwell at a density of 12 000 cells/well, whereas T98G glioma cells were seeded at a density of 10 000 cells/well. After 24 h, cells were treated with CBD at the indicated concentrations for 24 h. At the end of the incubation with the drug, MTT (0.5 mg/ml final concentration) was added to each well and the incubation was continued for further 4 h. The insoluble formazan crystals were solubilized by the addition of 100 µl of 100% dimethyl sulfoxide. Plates were read at 570 nm, using an automatic microtiter plate reader and data were expressed as the absorbance of the treated cells/control cells × 100.

### Invasion Assay

BD BioCoat matrigel invasion chambers were used to examine the ability of U87-MG and T98G cells to penetrate the ECM. 2.5×10^4^ cells were re-suspended in 500 µl of serum-free medium in presence of CBD, and added to the upper chamber. The lower chamber was filled with 0.75 ml of complete medium as chemo-attractant. Cells were then incubated for 24 h at 37°C. After removal of cells on the upper surface of the membrane, cells on the lower surface were fixed in 100% methanol and stained with Diff-Quick stain (Medion Diagnostics). Sixteen fields of cells were counted randomly in each well under a light microscope at 200× magnification. Data were expressed as the percentage of invasive cells as compared with the control.

### Human Array Kit/Proteome Profiler

To analyze the expression profiles of tumor-related proteins we used the Proteome Profiler™ Human Antibody Array Kit (R&D Systems), according to the Manufacturer’s instructions. This kit uses an array of 55 specific antibodies directed at proteins involved in tumor, angiogenesis and invasiveness, spotted onto a nitrocellulose membrane. Supernatants of CBD-treated and untreated U87-MG and T98G cells were centrifuged and mixed with 15 µl of biotinylated detection antibody for 1 h at room temperature. Then, the membranes were incubated with the sample/antibody mixtures overnight at 4°C on a rocking platform. Following a washing step to remove unbound material, streptavidin–horseradish and chemiluminescent detection reagents were added sequentially. Data on developed X-ray film were quantified by scanning on a transmission-mode scanner and analyzing the array image file using ImageJ analysis software. An averaged signal from the positive controls of each membrane was subtract from each protein spot.

### Western Blot Analysis

Cells were scraped and collected by centrifugation. Pellets were resuspended in lysis buffer (50 mM Tris-HCl, pH 7.4, 150 mM NaCl, 1 mM phenylmethyl-sulphonylfluoride, 10 mM NaF, 2 mM Na-orthovanadate, 1 mM EDTA, 1 mg/ml leupeptin, 1 mg/ml aprotinin, 1% Triton X-100, 0.1% SDS) in the presence of a protease inhibitor cocktail (Sigma Aldrich), at 4°C for 30 min. The protein content was determined according to BCA assay (Pierce) and equal amounts of proteins were separated on 14% SDS-polyacrylamide gel electrophoresis for TIMP-4 detection, on 12% gel for p-ERK and p-Akt detection and on 8% gel for MMP-9 and HIF-1α detection followed by electroblotting onto nitrocellulose membranes. The membranes were blocked with 5% milk in Tris-buffered saline/Tween-20 for 1 h and then probed overnight with the following antibodies: polyclonal antibody anti-MMP-9, N-terminus (MMP-9, 1∶2 000, Millipore), polyclonal antibody anti-TIMP4 (TIMP-4, 1∶1 000, Abcam), polyclonal antibody anti-phospho-p44/42 MAPK (Erk1/2) (Thr202/Tyr204) (p-ERK, 1∶500, Cell Signaling), polyclonal antibody anti-phospho-Akt (Ser473) (p-Akt, 1∶1 000, Cell Signaling), monoclonal antibody anti-human HIF-1α (1∶300, BD Biosciences), polyclonal antibody anti-ERK 1 (K-23) (total-ERK, 1∶3 000, Santa Cruz Biotechnology), polyclonal antibody anti-pan-Akt (pan-Akt, 1∶500, Abcam), monoclonal antibody anti-β-Actin (1∶5 000, Sigma Aldrich). Immunoreactive proteins were detected by incubation with horseradish peroxidase-conjugated IgG using the enhanced chemiluminescence system (ECL, Amersham). Signals were quantified by scanning on a transmission-mode scanner and analyzing the array image file using ImageJ analysis software.

### HIF-1α Stabilization and Hypoxia Induction

To achieve HIF-1α stabilization, two different methods were carried out: 1) cells were exposed to 50 µM CoCl_2_ for 24 h, a condition that mimics hypoxia by inhibiting prolyl hydroxylation of the oxygen-dependent degradation domain (ODDD) of HIF-1α and its subsequent interaction with the tumor suppressor protein Von Hippel Lindau; 2) cells were placed for 24 h into a modular incubator chamber (Billups Rothenberg) flushed with a mixture of 1% O_2_, 5% CO_2_ and 94% N_2_ at 37°C.

### Statistical Analysis

Statistical analysis was undertaken using GraphPad Prism 4.0 (GraphPad Software). All the experiments were performed in triplicates and results were expressed as mean ± S.E.M. of three independent experiments. One-way analysis of variance (ANOVA) followed by post-hoc analysis Dunnet’s t-test was used to evaluate statistical differences.

## Results

### Effect of CBD on Glioma Cell Invasion

**Figure 1:**
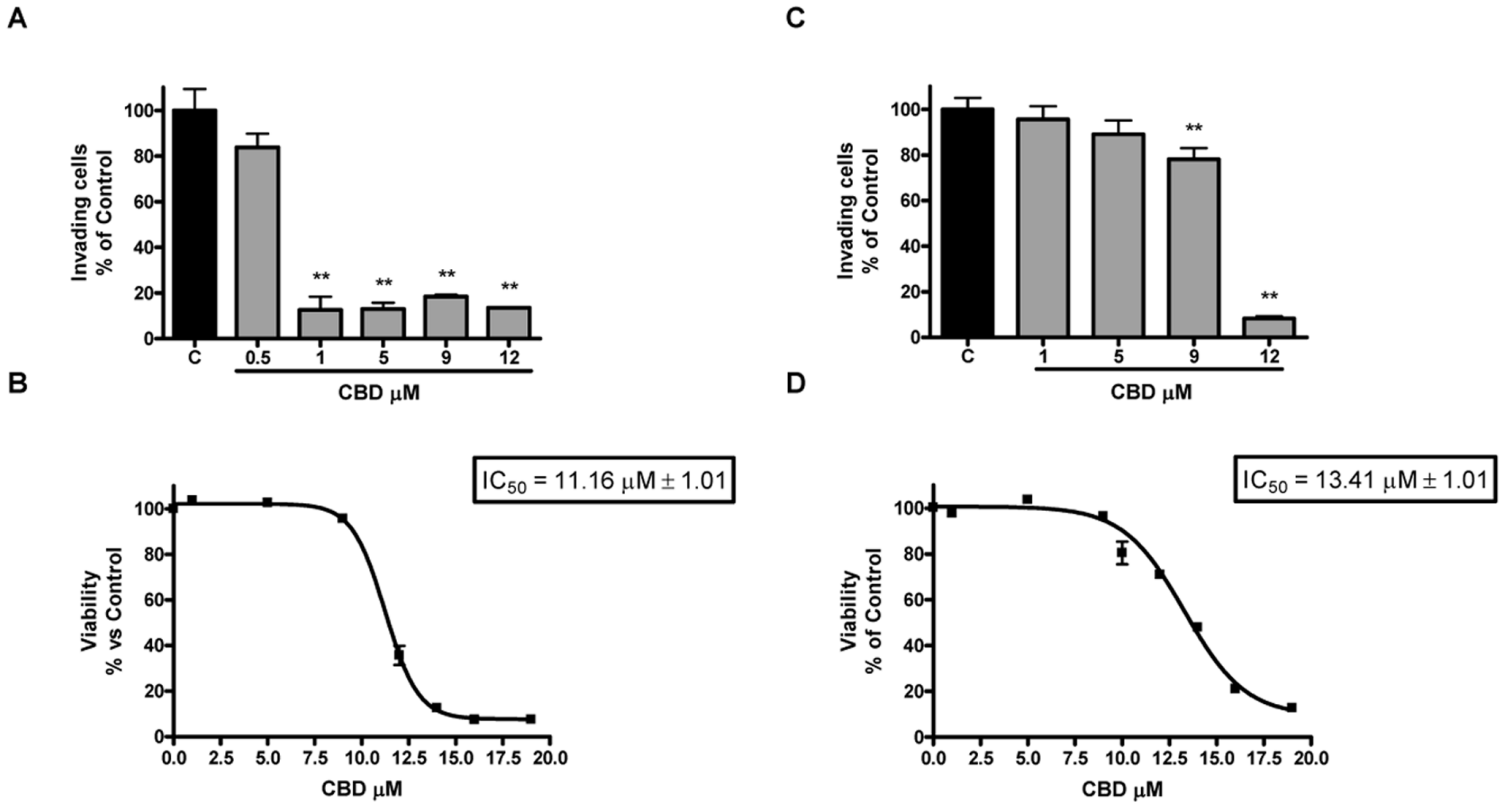
Effect of increasing CBD concentrations on U87-MG (A) and T98G (C) glioma cell invasion. U87-MG and T98G cells were treated with CBD, seeded onto a filter coated with matrigel and incubated for 24 h at 37°C. Cells that invaded the lower surface of the filter were quantified. The invasion was expressed as percentage of the untreated control. Data represent the mean ± S.E.M. of three independent experiments. **p<0.01 *vs* Control [C], Dunnett’s t test. Effect of increasing CBD concentrations on U87-MG (B) and T98G (D) glioma cell viability. U87-MG and T98G glioma cells were cultured in serum-free medium with increasing concentrations of CBD. Cell viability was determined by MTT assay after 24 h of treatment. The viability was expressed as percentage of the untreated control. Data represent the mean ± S.E.M. of at least three independent experiments.

The matrigel invasion assay was carried out to examine the effect of CBD on the invasiveness of U87-MG and T98G glioma cells. As shown in Fig. 1-A, CBD treatment caused a decrease of U87-MG cell invasion from 10% to 90% in the range of 0.5–12 µM, reaching the statistical significance at 1 µM. Corresponding experiments addressing a possible impact of CBD on cellular viability demonstrated that the anti-invasive concentrations were far from those causing toxic effects in the cells (MTT test, IC_50_ 11.16 µM; confidence limits 10.94 µM–11.38 µM; Fig. 1-B). When the experiments were performed in T98G cells (Fig. 1-C), CBD induced a slight, but significant, decrease of cell invasiveness at 9 µM (20%). Conversely, the highest concentration used, 12 µM, caused a strong reduction of the invasion (90%), though corresponding only to a 30% decrease in cell viability, as assessed through MTT assay (IC_50_ 13.41 µM; confidence limits 13.09 µM–13.86 µM; Fig. 1-D).

### CBD Modifies the Expression Pattern of Tumor-related Proteins in Glioma Cells

**Figure 2:**
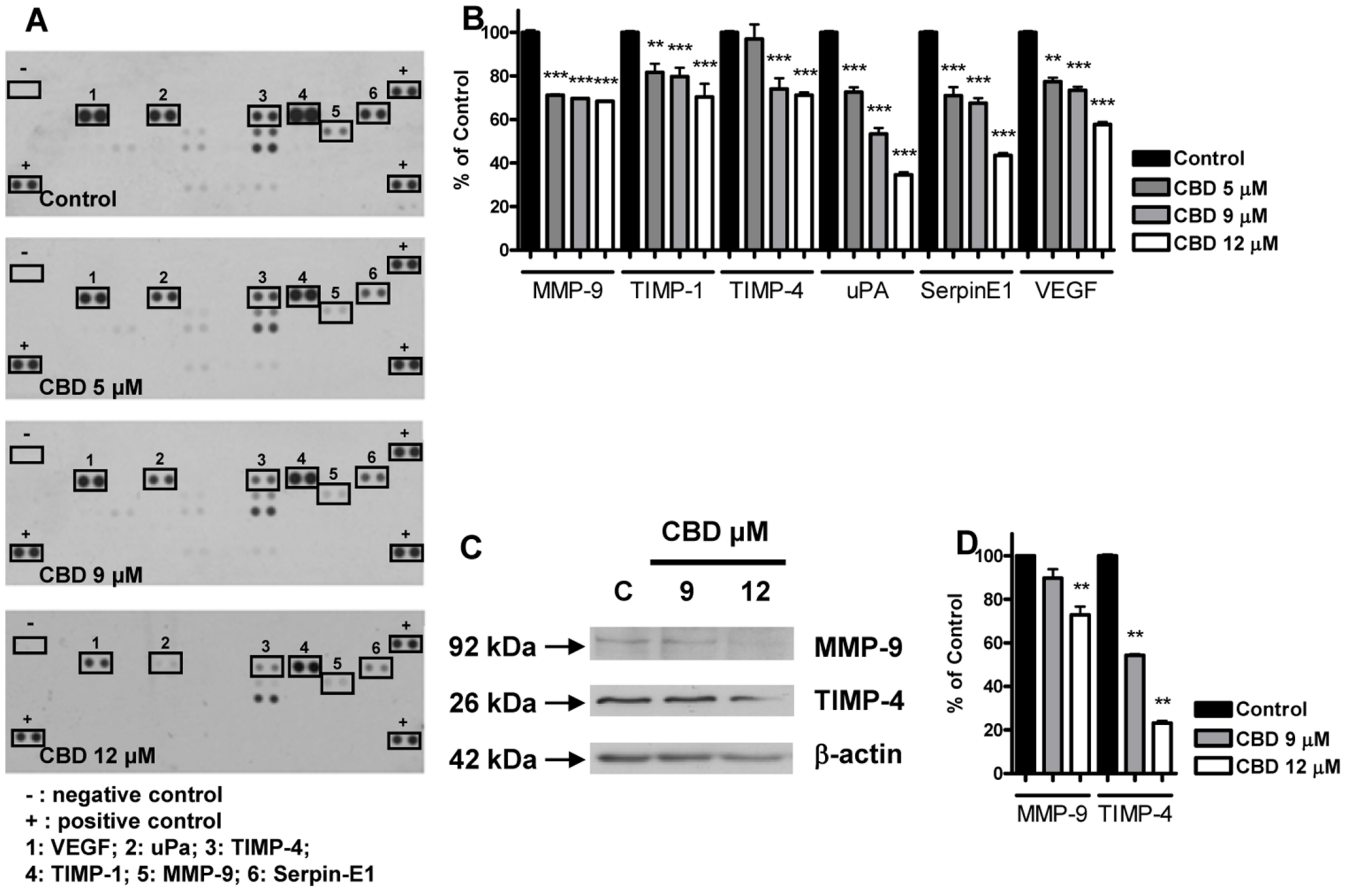
Effect of increasing CBD concentrations on the protein expression profile of U87-MG glioma cells. U87-MG cells were treated with CBD for 24 h and supernatants were used to determine different protein levels through a human array kit/proteome profiler. (A) Representative proteomic membrane analysis with indication of the modified proteins. (B) Densitometric analysis of the membrane spots reported as percentage of the untreated control. Data represent the mean ± S.E.M. of three independent experiments. **p<0.01, ***p<0.001 *vs* Control, Dunnett’s t test. U87-MG cells were treated with CBD for 24 h and lysates from cells were used to assess MMP-9 and TIMP-4 protein levels. (C) Western blot analysis of MMP-9 and TIMP-4. A representative western blot is shown. (D) Densitometric analysis of MMP-9 and TIMP-4 signal bands from three independent experiments is reported. **p<0.01 *vs* Control [C], Dunnett’s t test.

**Figure 3:**
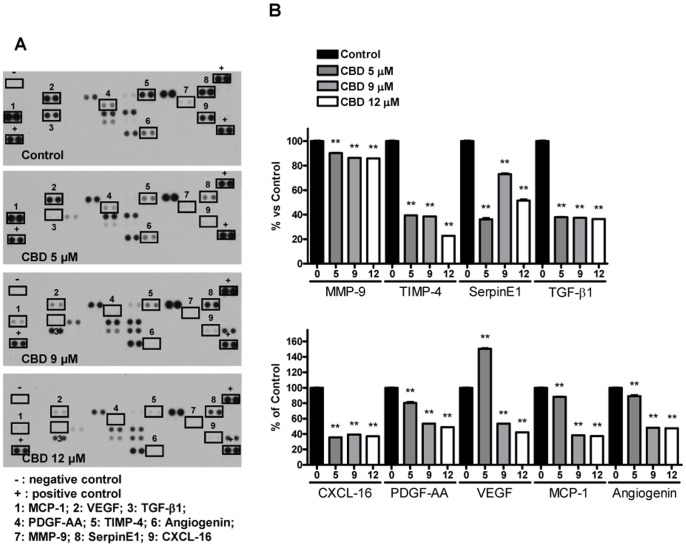
Effect of increasing CBD concentrations on the protein expression profile of T98G glioma cells. T98G cells were treated with CBD for 24/proteome profiler. (A) Representative proteomic membrane analysis with indication of the modified proteins. (B) Densitometric analysis of the membrane spots reported as percentage of the untreated control. Data represent the mean ± S.E.M. of three independent experiments. **p<0.01 *vs* Control, Dunnett’s t test.

Since tumor cell growth, invasion and angiogenesis depend on complex pathways and activity of different proteins, we analyzed whether CBD interfers with the expression profile of a set of proteins involved in tumor development in glioma cells, using a rapid and sensitive antibody array-based assay. The array images reported in Fig. 2-A and 3-A allow a qualitative assessment of the effect of CBD on the expression pattern of multiple proteins released by U87-MG and by T98G cells and captured by the specific pre-spotted antibodies on nitrocellulose membranes (see Materials and methods). Among all the detectable spots, CBD down-regulated six proteins in U87-MG cells, namely matrix metalloproteinase MMP-9, tissue inhibitors of metalloproteinase TIMP-1 and TIMP-4, urokinase plasminogen activator (uPA), SerpinE1-plasminogen activator inhibitor type-1 (*PAI*-1), and VEGF. The extent of the downregulation was different depending on the protein, ranging from 20% up to 60% compared to the control (Fig. 2-B). Regarding T98G cells, the panel of inhibited proteins only partially overlaps with the one obtained for U87-MG cells. In addition to the downregulation of MMP-9, TIMP-4, SerpinE1-PAI-1 and VEGF shown in U87-MG cells, in T98G cells CBD treatment also caused a significant reduction in the level of TGF-β1, CXCL-16, PDGF-AA, MCP-1 and Angiogenin. The inhibition ranged from 12% up to 80% compared to the control, depending on the considered protein (Fig. 3-B). The data of proteome analysis were confirmed in U87-MG cells by western blot analysis on MMP-9 and TIMP-4 (Fig. 2-C,-D). Consistent with the data obtained by the array CBD significantly reduced MMP-9 and TIMP-4 levels in a concentration-dependent way.

### Identification of Signaling Pathways Modulated by CBD in Glioma Cells

**Figure 4:**
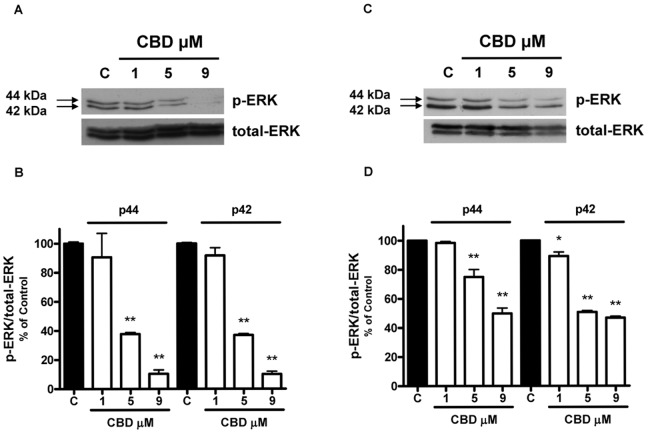
Effect of increasing CBD concentrations on ERK phosphorylation in U87-MG and T98G glioma cells. U87-MG and T98G cells were treated with CBD for 24 h and lysates from cells were used to assess ERK protein levels. (A,C) Western blot analysis of phospho- and total-ERK. A representative western blot is shown. (B,D) Densitometric analysis of ERK signal bands from three independent experiments is reported. *p<0.05, **p<0.01 *vs* Control [C], Dunnett’s t test.

**Figure 5:**
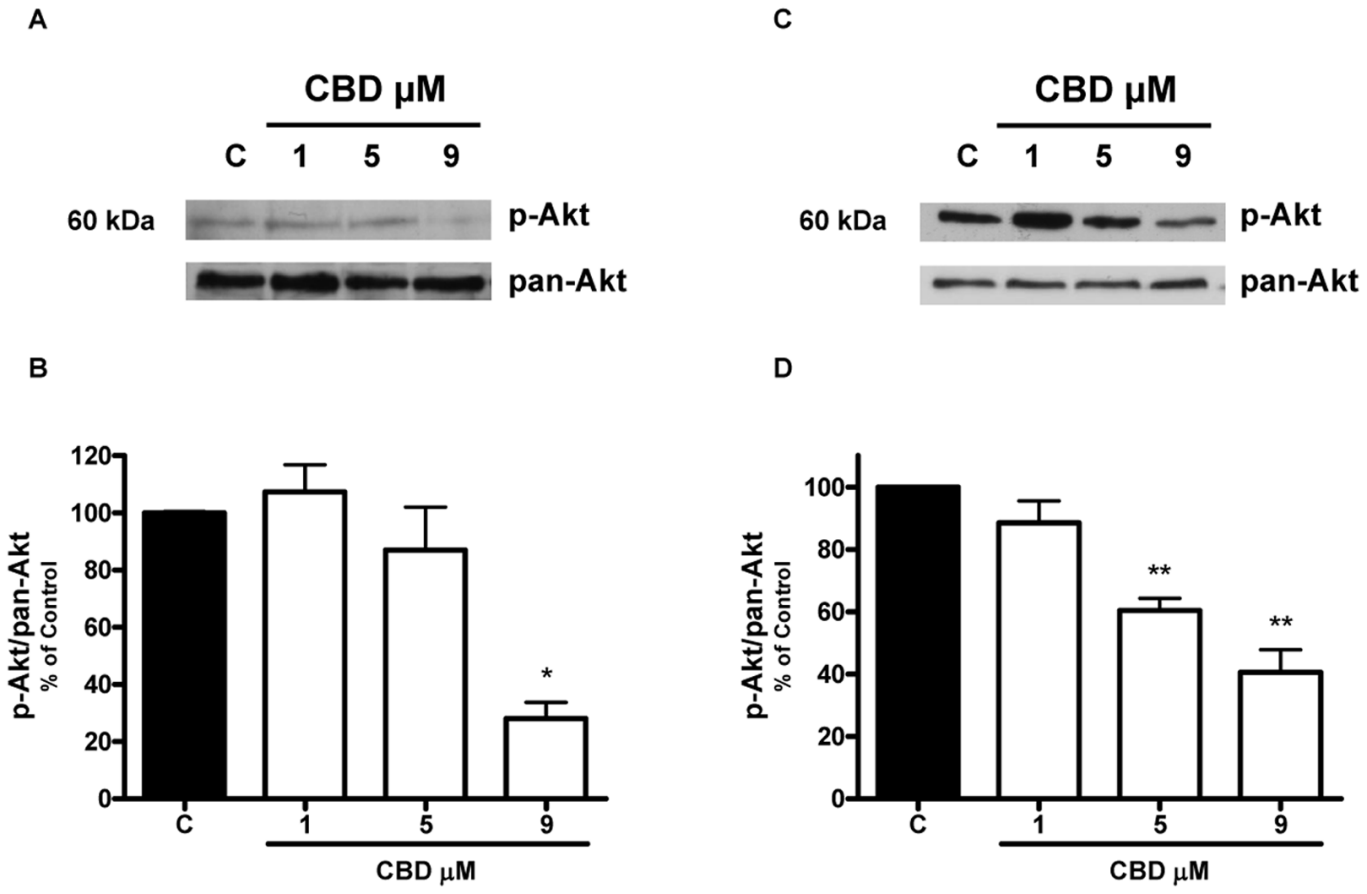
Effect of increasing CBD concentrations on Akt phosphorylation in U87-MG and T98G glioma cells. U87-MG and T98G cells were treated with CBD for 24 h and lysates from cells were used to assess Akt protein levels. (A,C) Western blot analysis of phospho- and pan-Akt. A representative western blot is shown. (B,D) Densitometric analysis of Akt signal bands from three independent experiments is reported. *p<0.05, **p<0.01 *vs* Control [C], Dunnett’s t test.

Since modulation of the kinase activity of ERK1/2 and Akt has been reported to play an important role in the tumorigenicity of glioma cells [14]–[16], we performed western blot analysis aimed at examining the effect of CBD on the phosphorylation status of these two kinases. As shown in Fig. 4, the exposure of U87-MG and T98G cells to CBD caused a dose-related reduction in the levels of ERK1/2 phosphorylated forms, whereas total ERK protein level was unaffected. Similarly, in both cell lines CBD also reduced constitutive Akt phosphorylation, with no effect on total Akt protein level (Fig. 5).

### Effect of CBD on HIF-1α Expression in Glioma Cells

**Figure 6:**
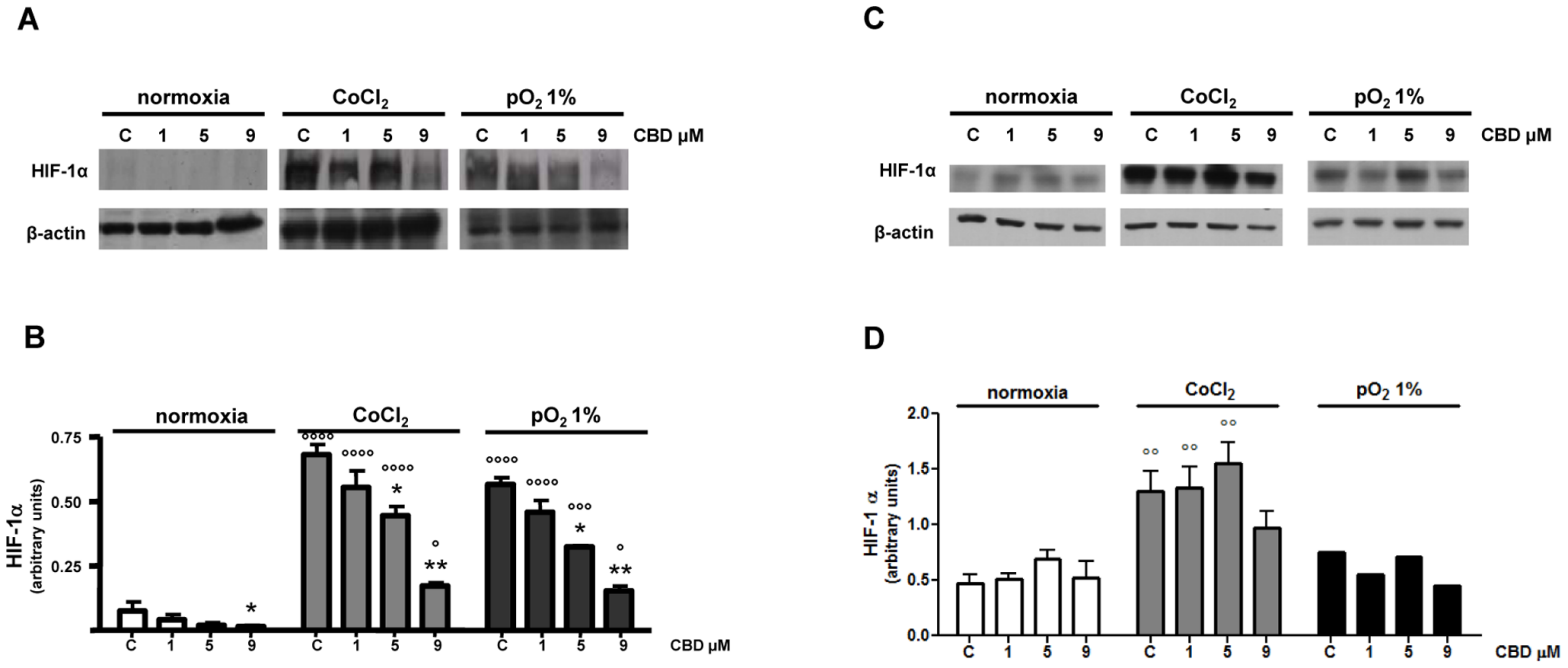
Effect of CBD on HIF-1α level in glioma cells grown in normoxic and hypoxic conditions. U87-MG and T98G glioma cells were grown under normoxia and two different hypoxic conditions (see Materials and methods). Cells were treated with CBD for 24 h and lysates from the cells were used to assess HIF-1α protein levels. (A–C) Western blot analysis of HIF-1α and β-actin. A representative western blot is shown. (B–D) Densitometric analysis of HIF-1α signal bands from three independent experiments is reported. *p<0.05, **p<0.01 *vs* Control [C], ° p<0.05, °° p<0.01, °°° p<0.001, °°°° p<0.0001 *vs* normoxia Control [C], Dunnett’s t test.

As several aspects of tumor growth and invasiveness depend on the transcriptional activation of diverse sets of genes regulated by HIF-1, the hypothesis that CBD antitumor effect might be accompanied by the modulation of HIF-1α expression was tested in U87-MG and T98G cells under experimental conditions that cause HIF-1α stabilization, namely exposure to 50 µM CoCl_2_ or 1% pO_2_ for 24 h. Exposure of U87-MG cells to CoCl_2_ or to 1% pO_2_ induced a marked increase of HIF-1α levels (Fig. 6-A), as compared to normoxic cells, where HIF-1α protein was barely detectable. Under both simulated and actual hypoxic conditions, CBD at 5 and 9 µM concentrations was found to induce a significant decrease in HIF-1α levels, as demonstrated by the densitometric analysis of the protein (Fig. 6-B). Differently from U87-MG cells, HIF-1α protein was low, but present in normoxic T98G cells (Fig. 6-C,-D). Exposure of T98G cells to CoCl_2_ determined a significant increase in HIF-1α levels, as compared to normoxic cells, whereas exposure to 1% pO_2_ caused no significant difference (Fig. 6-C). In all the considered conditions CBD induced no significant change in HIF-1α level, as demonstrated by the densitometric analysis of the protein (Fig. 6-D). Interestingly, CBD showed similar antiproliferative effects in both hypoxic conditions and under normoxic state (data not shown).

## Discussion

Results presented herein demonstrate that the non psychoactive phytocannabinoid CBD inhibits U87-MG and T98G cell invasion and proliferation, and shed light on the molecular mechanisms that drive its anti-tumoral action. The high rate of tumor cell invasiveness into normal brain tissue is a defined hallmark of gliomas and represents an important aspect accounting in large part for their poor prognosis. The ability of tumoral cells to invade the normal tissue is crucial for tumor spread, growth, and metastasis. Thus the ability of anticancer drugs to induce inhibition of cell invasion, besides apoptosis and/or cell growth arrest, is considered fundamental to the success of cancer therapy. Our results demonstrate that CBD effectively inhibited glioma cell invasion, though with different potency in the two different cell lineages considered, as tested by the matrigel Boyden assay. Interestingly, as we already demonstrated for migration [12], the decreased invasiveness elicited by CBD treatment was significant at concentrations lower than those required to cause 50% inhibition of viability and proliferation (1 µM vs 11 µM in U87-MG cells, 9 µM vs 13 µM in T98G cells, MTT assay run in parallel to the invasion test). The ability of CBD to reduce glioma invasiveness is in accordance with the data of Marcu et al. [17] on other glioma cell lines, although the range of effective concentrations and cell sensibility appear different in the two studies. This may be due to the different experimental conditions applied (3-days treatment in Marcu’s paper vs 24 hours of exposition in ours) and to the cell lines used (SF126 and U373 cell lines vs U87-MG and T98G).

In the invasion assay, CBD does not seem to show a classic dose-dependent response, showing a very narrow dose range. This could be related to the fact that CBD has been previously reported to act on gliomas through a cannabinoid and vanilloid receptor-independent mechanism [9], [12], [18]. Therefore, the lack of a classic dose-dependent effect of CBD in our matrigel experiments could be partially related to its low affinity for cannabinoid and vanilloid receptors. Moreover, other CBD pharmacological effects tested in various models are not always dose-related [19], [20] and even some other structure-related cannabinoids, such as HU-336 (tetrahydrocannabinol quinone) and HU-345 (cannabinol quinone), were able to inhibit aortic ring angiogenesis without a clear dose-response relationship [21].

A second highlight of the present work is the evidence that active CBD concentrations produce a widespread inhibition of several proteins involved in cell growth and invasion in both cell lines.

In U87-MG cells we found a sustained inhibition of MMP-9, TIMP-1, TIMP-4, uPA, SerpinE1-PAI-1 and VEGF. All these proteins are differently involved in malignancy, motility, invasion and angiogenesis. MMPs are fundamental proteases up-regulated in gliomas and strictly associated with the malignancy of these tumors [22]. Indeed they favour tumor cell motility, invasion and angiogenesis by degrading ECM and act as chemotactic signals [23]. Therefore, MMP-9 inhibition can represent part of the mechanism through which CBD efficiently slows down the invasive feature of U87-MG cells. In addition, we found inhibition of TIMP-1, a stromal factor with multiple functions, whose over expression correlates with aggressive clinical behavior of a spectrum of tumors, gliomas included. Even though Ramer’s work demostrated CBD-driven TIMP-1 upregulation and consequent decrease of invasion in cervical and lung cancer cells, this discrepancy is likely due to the different kind of tumor considered [24]. TIMP-1 is also reported to be a target of MAPKs [24], [25] and, on the other hand, a signaling factor able to stimulate ERK and Akt pathways resulting in cell proliferation [26]. Thus, the decrease in TIMP-1 fits well with the decrease in ERK and Akt that we found after CBD treatment. In contrast, the observed decrease of TIMP-4 level is difficult to explain and reconcile with CBD antitumor effects, since the only study addressing the role of TIMP-4 in gliomas reported a negative correlation between TIMP-4 level and the grade of the disease [27]. Further studies are needed to clarify the involvement of TIMPs in brain tumor progression.

Besides the MMP/TIMP system, we found significant alteration in the urokinase plasminogen activator uPA and in the plasminogen activator inhibitor SerpinE1-PAI-1, two important factors in the regulation of cancer cell growth and spreading. uPA participates in cancer cell invasiveness and its inhibition correlates well with CBD action, whereas the reduction in SerpinE1-PAI-1 level is more difficult to explain. Indeed low levels of this protein would be expected to favour cancer cell growth, but some clinical studies have indicated that SerpinE1-PAI-1 expression is strongly related with poor outcome in cancer. At present, this apparent discrepancy is not understood and some recent reports point to other multifunctional roles of the protein in angiogenesis, invasiveness and cell adhesion [28]. Finally, we found that CBD significantly inhibited VEGF, one of the most potent stimuli in angiogenesis able to drive the growth of new blood vessels.

Regarding T98G cells, the panel of inhibited proteins only partially overlaps with the one obtained for U87-MG cells. In fact, in addition to the downregulation of MMP-9, TIMP-4, SerpinE1-PAI-1 and VEGF, in T98G cells CBD treatment caused a significant reduction in the level of TGF-β1, CXCL-16 and PDGF-AA, promotors of cell growth and invasion [29]–[32]. It also reduced the level of MCP-1, a member of the cytokine/chemokine superfamily. MCP-1 is specifically secreted by glioma cells to inhibit antitumor immune responses and facilitate tumor growth [33], [34]. Finally, CBD determined a decrease in the potential angiogenic factor Angiogenin, whose increase significantly correlates with the higher grade of glioma malignancy [35].

These results suggest that CBD efficacy in inhibiting tumor growth and invasion proteins shows differences between the two considered cell lines, despite a similar potency in reducing cell viability. This may reside in the different genetic background of the two lines due to the different brain tumor subtype that they were isolated from. In fact, U87-MG cell line derives from glioblastoma/astrocytoma, whereas T98G from glioblastoma multiforme. Moreover U87-MG cells are well known to respond to cannabinoid treatment, whereas T98G cells have been so far considered resistant to cannabinoids, based on their insensitivity to Δ^9^-THC [36]. It is worth noting that CBD, differently from Δ^9^-THC, seems to interfere with growth and invasion even in T98G cells.

Besides the wide inhibition of different proteins, CBD strongly down-regulated two signaling pathways critical for glioma cell survival and proliferation, such as ERK and PI3K/Akt. These signaling molecules have been shown to play a crucial role in tumor cell proliferation and in cell escape from apoptosis or cycle arrest as well as in cell motility and invasion [37], [38]. Similar results were recently obtained by Soroceanu et al. (2013) in U251 cells, demonstrating a CBD-dependent ERK and Akt downregulation in glioma cells, together with a decrease in MMP-2 level and in invasiveness. They suggest that CBD may act through modulation of Id-1, a transcription factor regulator that seems to be involved in glioma malignancy [39].

Moreover, it has been demonstrated that MMP activation is highly regulated by MAPK activation [40]. In addition, Akt is highly activated in gliomas and represents a nodal point in cell signaling stimulated by several upstream stimuli, including growth factors [14], [16]. Thus, in human malignancies, kinases represent an attractive target for anticancer research and numerous kinase inhibitors are currently under investigation in human clinical trials [41], [42]. Therefore, simultaneous pharmacological inhibition of both Akt and ERK by CBD might represent a valuable effect for its potential therapeutic use in gliomas. Recently, Marcu et al. [17] reported that treatment with CBD alone induced no changes in ERK phosphorylation in human glioblastoma cells. This discrepancy is only apparent, since they used a very low CBD concentration (0.4 µM). Accordingly, also in our experiments CBD has no effect at 1 µM concentration, the inhibition being present from 5 µM and above.

Finally, another important outcome of this research is the demonstration for the first time that, al least in U87-MG cells, CBD inhibits HIF-1α, the regulatory subunit of the hypoxia-inducible transcription factor. Gliomas have extensive areas of severe hypoxia and necrosis, which represent a major obstacle to the success of chemotherapy. HIF-1α is one of the major factors responsible for orchestrating the adaptive transcriptional programs, inducing cell survival, motility and tumor angiogenesis under hypoxic conditions [6], [43]. Thus, CBD inhibition of HIF-1α can represent, at least in U87-MG cells, one of the aspects through which the drug exerts its significant antineoplastic activity. On the other hand, in T98G cells HIF-1α level is already high in normoxic conditions, whereas in U87-MG cells, as expected, the protein is barely detectable. This suggests that upstream pathways responsible for HIF-1α activation are likely to be different in the two cell lines, those evoked by T98G cells being mainly insensitive to CBD treatment.

We still do not know the precise mechanism of action of CBD. Previous results have already excluded any involvement of cannabinoid and vanilloid receptors due to the low affinity of CBD to both CB1 and CB2 receptors [15] an to TRPV1 receptor [44]. GPR55 is an orphan G-protein linked receptor that appears up-regulated in some cancer-derived cell lines and plays a pivotal role in tumor cells [45]. Recently, it has been proposed that GPR55 might account for some of the non-CB1, non-CB2 cannabinoid effects [46]. However, at the moment, the possible involvement of GPR55 and its precise role in mediating CBD effects is still controversial. Future experiments using siRNA against GPR55 will help to clarify the role of this receptor in CBD anticancer activity.

In conclusion, the present investigation confirms the antiproliferative and antiinvasive effects of CBD in U87-MG cells. More interestingly, these effects can also be extended to T98G glioma cells, a well known Δ^9^-THC-resistant lineage. Moreover, our data add further insights into CBD antitumor action, showing its ability to influence multiple cellular targets in the two cell lines with partial overlap. Future studies are urgently needed to highlight the molecular mechanism through which CBD can influence the different intracellular pathways taken into account in the present work.
